# Metabolizable and Net Energy Values of Corn from Various Regions and Prediction Equation Development for Arbor Acres Broilers at Two Growth Stages

**DOI:** 10.3390/ani16182816

**Published:** 2026-09-08

**Authors:** Xunyu Guo, Lijia Li, Shuhang Yin, Zhibin Ban, Wei Nie

**Affiliations:** 1 State Key Laboratory of Animal Nutrition and Feeding (SKLANF), College of Animal Science and Technology, China Agricultural University, Beijing 100193, China; 2 Jilin Academy of Agricultural Sciences (Northeast Agricultural Research Center of China), Changchun 130033, China

**Keywords:** corn, AA broilers, available energy values, prediction equations

## Abstract

This study aimed to determine the apparent metabolizable energy (AME), nitrogen-corrected AME (AMEn), and net energy (NE) values of corn for Arbor Acres (AA) broilers aged 12–14 and 26–28 days and to develop prediction equations. Ten corn samples with varying chemical compositions were selected. Test diets were formulated using the substitution method, in which corn replaced 40% of the energy-supplying components in a basal diet. A total of 396 male AA broilers at 9 days of age and another 396 at 23 days of age were used across the two metabolism trials. AME, AMEn and NE were determined using the substitution method combined with respiratory calorimetry. Correlations between energy values and chemical components were analyzed. Multiple linear stepwise regression in SPSS was used to establish prediction equations for AME, AMEn, and NE. The results showed that for 12–14-day-old broilers, AME, AMEn, and NE ranged from 13.84–15.66 MJ/kg, 13.39–15.35 MJ/kg, and 8.61–12.56 MJ/kg, respectively. For 26–28-day-old broilers, the corresponding values ranged from 14.62–16.30 MJ/kg, 14.42–16.05 MJ/kg, and 10.42–13.06 MJ/kg. Notably, NE values and AME/GE (gross energy) ratios were significantly higher in 26–28-day-old broilers than in 12–14-day-old broilers (*p* < 0.05), which increased by 9.33% and 3.96%, respectively. Correlation analysis showed that phytic acid (PA) was significantly negatively correlated with AME, AMEn, NE, AME/GE, and AMEn/GE in the younger group, whereas ash content was significantly negatively correlated with AME and AMEn in the older group. The prediction equations exhibited good fit, with the coefficient of determination (R^2^) of the best equation reached 0.97. These findings support the precise application of corn in feed formulation and provide a scientific basis for its efficient utilization in poultry production.

## Simple Summary

Corn is the primary energy source in poultry feed, but its energy content can vary greatly depending on variety and growing conditions. Accurately knowing how much energy corn provides is essential for formulating cost-effective and nutritious diets for broiler chickens. In this study, we measured the energy values of ten different corn samples from various regions using two groups of broilers at different ages (12–14 days and 26–28 days). We found that energy values varied considerably among corn samples and that older broilers were able to utilize energy from corn more efficiently than younger broilers. We also identified that certain chemical components in corn, such as phytic acid and ash content, were linked to lower energy availability. Based on these results, we developed mathematical formulas that can predict the energy value of corn from simple chemical analysis, without the need for time-consuming and expensive animal feeding trials. These prediction tools can help feed manufacturers and farmers quickly estimate corn quality, optimize feed formulations, reduce costs, and improve the efficiency and sustainability of poultry production.

## 1. Introduction

Feed costs account for more than 60% of total production costs in poultry production, and energy is one of the most critical parameters in feed formulation [1]. Accurate evaluation of the available energy values of feed ingredients is essential for precision nutrition and cost control. Traditional animal trial methods are time-consuming and expensive, making them inadequate for the rapid, batch-wise assessment of ingredient energy values required by the modern feed industry [2]. Therefore, developing rapid and accurate prediction equations based on proximate chemical composition is of great significance. Currently, the metabolizable energy (ME) system is widely adopted in poultry evaluation. However, the ME system may underestimate the energy of dietary fat and overestimate that of protein [3]. The net energy (NE) system, defined as apparent ME minus heat increment (HI) [4], accounts for heat production during digestion and represents the energy truly available for maintenance and production [5]. De Groote et al. [3] reported that the NE system outperforms the ME system in broiler diet formulation, as it can reduce diet costs and improve economic returns. Consequently, establishing an accurate NE system and reliable prediction equations is critically important.

In current intensive broiler production, corn–soybean meal diets are the most common type [6]. Corn serves as the primary energy source, with inclusion rates much higher than those of wheat, barley, or sorghum [7]. This preference is mainly attributed to its high starch content and low non-starch polysaccharide (NSP) content [8]. Although many studies have investigated corn energy values, most have focused on determining ME and developing ME prediction equations [9,10].

Therefore, this study aimed to determine the apparent metabolizable energy (AME), nitrogen-corrected AME (AMEn), and NE values of corn and to establish prediction equations for available energy values in Arbor Acres (AA) broilers. We hypothesized that (1) corn from different sources varies substantially in AME, AMEn, and NE values; (2) broiler age improves energy utilization efficiency; and (3) reliable prediction equations can be derived from routine chemical composition. The results provide a theoretical basis for estimating the energy values of unknown corn samples using chemical composition, thereby supporting the precise application and scientific formulation of corn in broiler diets.

## 2. Materials and Methods

### 2.1. Animal Welfare Statement

The Animal Ethics Committee of China Agricultural University approved this study (Approval No. Aw161706202-1-01). All animal procedures were carried out strictly following the Regulations for the Administration of Experimental Animals, which were formulated by the Ministry of Science and Technology (Beijing, China).

### 2.2. Corn and Diets

Ten corn samples (Corn 1 to Corn 10) were selected, and their chemical compositions are presented in Table 1. Corn 7 originated from the United States. The other samples were collected from various regions in China, including Ningxia (Corn 1), Shaanxi (Corn 2), Jilin (Corn 3 and Corn 5), Henan (Corn 4), Inner Mongolia (Corn 6), Liaoning (Corn 8), Xinjiang (Corn 9), and Shandong (Corn 10). These samples were deliberately chosen from seven geographically diverse corn-producing regions in China and the United States, spanning a wide range of climatic conditions and cultivation practices, to capture the full spectrum of chemical variability typically observed in commercial production. Indeed, the coefficients of variation for most nutrients exceeded 10% (Table 1), confirming that these samples adequately represent the natural diversity encountered in practice. The experimental diets included one basal diet and ten test diets, with formulations and nutritional compositions shown in Table 2 and Table 3, respectively. All diets were formulated to meet the nutritional requirements of broilers according to the Chinese Feeding Standard of Chicken (NY/T 33-2004) [11]. The test diets were prepared using the substitution method, where each corn sample replaced 40% of the basal diet, following the procedure described by Guo et al. [12]. Vitamin premix, minerals (including calcium), and other non-energy components were adjusted to ensure identical inclusion rates across all diets.

### 2.3. Respiration Calorimetry Chambers

The open-circuit respiration calorimetry chambers were developed by the Poultry Energy Metabolism Research Team of the Jilin Academy of Agricultural Sciences. Each chamber was equipped with a remotely controlled lid. At the start of each trial, the lid was closed and sealed with water at the edges to ensure airtightness. The chambers were fitted with vacuum pumps, as well as CO_2_ and O_2_ sensors. Gases were continuously drawn from the chambers at a flow rate of 10 L/min. Gas concentrations at the inlet and outlet of each chamber were measured by analyzers over a 3-min period, with residual gases removed before each measurement. The O_2_ analyzer ranged from 0% to 25% (zirconia sensor, 65-4-20; Advanced Micro Instruments Inc., Huntington Beach, CA, USA), and the CO_2_ analyzer ranged from 0% to 2.5% (non-dispersive infrared sensor, AGM 10; Sensors Europe GmbH, Erkrath, Germany). Heat production (HP) and respiratory quotient (RQ) were calculated from CO_2_ production and O_2_ consumption [13].

### 2.4. Broilers and Management

Male AA broilers were obtained from a local hatchery. At two distinct ages (9 d and 23 d), 396 broilers were randomly allocated across 11 dietary treatments, with six replicates per treatment (six broilers per replicate). Within each replicate, four broilers were placed in conventional metabolism cages for excreta collection, and the remaining two were transferred to the respiration chambers for gaseous exchange recordings. At the start of each trial, the average body weight among replicates was balanced to within 3% of the overall mean. Broilers had free access to feed and water throughout the entire study.

The experiment followed a completely randomized design, with each diet being tested in six independent runs (one run per chamber per replicate). Each experimental period lasted 6 days: an initial 3-day acclimation phase for diet and environmental adaptation, followed by a 3-day data collection phase. During the collection window, feed intake was recorded daily per replicate, and HP was measured continuously over the same 3 days using the calorimetry system.

### 2.5. Sample Collection and Chemical Analyses

Excreta from metabolism cages were gathered at 24-h intervals (09:00 h) over the 72-h collection period. Prior to each collection, trays were cleared of feathers and scales. Collected excreta were immediately sprayed with 10% HCl to fix nitrogen and stored at −20 °C. After the trial, the 3-day pooled excreta per replicate were thawed, oven-dried at 65 °C for 72 h, and ground to pass through a 40-mesh screen. Similarly, corn, diet, and excreta samples were ground and preserved at −4 °C pending analysis.

Gross energy was determined with a C 2000 bomb calorimeter (IKA, Guangzhou, China), while proximate components (DM 934.01, CP 990.03, ash 920.39, CF 978.10, EE 942.05, ST 2002.02) were analyzed by AOAC methods [14]. Neutral and acid detergent fibers (NDF and ADF) were quantified using an Ankom 220 fiber analyzer (Ankom Technology, Macedon, NY, USA) according to Van Soest et al. [15]. Phytic acid (PA) in corn was measured with a commercial assay kit (ADS-W-QT017, Jiangsu Addison Biotechnology Co., Ltd., Nanjing, China).

### 2.6. Energy Value Calculation

The AME, AMEn and NE of each corn sample were derived using the substitution formula:

$${AME}_{ingredient}\;(\mathrm{MJ}/\mathrm{kg})=({AME}_{test\;diet}-{AME}_{reference\;diet}\times a\%)/b\%$$

$${AMEn}_{ingredient}(MJ/kg)=(AMEn_{test\;diet}-AMEn_{reference\;diet}\times a\%)/b\%$$

$${NE}_{ingredient}(\mathrm{MJ}/kg)=({NE}_{test\;diet}-{NE}_{reference\;diet}\times a\%)/b\%$$

The substitution level of the corn sample under investigation and the share of energy-supplying components originating from the basal diet in the test diet are denoted by b% and a%, respectively. Dietary energy values (AME_diet, AMEn_diet, and NE_diet) were calculated according to the method described by Mera-Zúñiga et al. [16].

### 2.7. Statistical Analysis

All statistical analyses were performed using SPSS 26.0 (IBM Corp., Armonk, NY, USA). Prior to one-way ANOVA, normality of the data was assessed using the Shapiro–Wilk test, and homogeneity of variances was evaluated using Levene’s test. All data met these assumptions, justifying the use of parametric tests. Statistical comparisons between two groups were made using Student’s *t*-test, while one-way ANOVA with Tukey’s HSD post hoc was performed for multiple group mean comparisons. Spearman’s rank correlation coefficient was utilized to examine relationships among variables. Data are expressed as means ± pooled standard error of the mean (SEM). Differences were deemed significant at *p* < 0.05. For predictive modeling, multiple linear stepwise regression was applied to generate equations for estimating corn energy values, and model performance was assessed using the coefficient of determination (R^2^) and residual standard error (RSE). Multicollinearity among predictor variables was diagnosed using the variance inflation factor (VIF), with VIF values < 10 for all variables retained in the final models, indicating no serious collinearity issues. It should be noted that the prediction equations were developed using only 10 corn samples and were not subjected to internal cross-validation or external validation with an independent dataset. Therefore, caution is warranted when extrapolating these equations to broader populations, and future studies with larger and more diverse corn samples are needed to further validate and refine the models.

## 3. Results

### 3.1. Chemical Composition of Corn

As shown in Table 1, the chemical composition of the 10 corn samples exhibited substantial variability across nutrient fractions. The coefficients of variation (CV) for most nutrients exceeded 10%. Among all components, ADF showed the highest variability (CV = 23.46%), whereas GE showed the lowest (CV = 0.91%), indicating that GE remained relatively constant across samples. DM and ST were also relatively stable, with CVs of 1.04% and 6.74%, respectively. The remaining components, including CP, EE, CF, NDF, Ash, and PA, had CVs ranging from 11.65% to 19.42%.

### 3.2. Energy Values and Energy Utilization Efficiency of Corn

For 12–14-day-old broilers (Table 4), AME, AMEn and NE varied considerably among corn samples. Corn 5 had the highest AMEn, which was significantly higher than that of Corn 3 (*p* < 0.05), but did not differ significantly from other samples. For NE, Corn 1 had the highest value, while Corn 9 had the lowest; however, no significant differences were detected among samples (*p* = 0.062).

Regarding energy utilization efficiency in 12–14-day-old broilers, the mean values of AME/GE, AMEn/GE, NE/AME, and NE/AMEn were 79.31%, 77.93%, 72.53%, and 73.87%, respectively. Corn 5 had the highest AMEn/GE, significantly higher than Corn 3 (*p* < 0.05). Corn 3 had the lowest AME/GE and AMEn/GE. For NE/AME and NE/AMEn, Corn 1 showed the highest conversion efficiencies, whereas Corn 9 showed the lowest.

For 26–28-day-old broilers (Table 5), AME and AMEn also varied among samples. Corn 9 had the highest AME and AMEn, which were significantly higher than those of Corn 4 (*p* < 0.05), but did not differ from other samples. No significant differences in NE were observed among corn samples (*p* > 0.05).

For energy utilization efficiency in 26–28-day-old broilers, the mean values of AME/GE, AMEn/GE, NE/AME, and NE/AMEn were 82.45%, 80.66%, 76.68%, and 78.38%, respectively. Corn 9 had the highest AME/GE and AMEn/GE, significantly higher than Corn 4 (*p* < 0.05), but not significantly different from other samples (*p* > 0.05). Corn 7 showed the highest NE/AME and NE/AMEn, while Corn 3 showed the lowest, but no significant differences were detected among groups for these two parameters (*p* > 0.05).

Furthermore, as shown in Table 6, the NE values of 12–14-day-old broilers were significantly lower than those of 26–28-day-old broilers (*p* < 0.05). In terms of energy utilization efficiency, the AME/GE ratio was significantly higher in 26–28-day-old broilers (*p* < 0.05), and the AMEn/GE ratio showed an increasing trend (0.05 < *p* < 0.1).

### 3.3. Correlation Analysis and Prediction Equations

Correlation analyses between corn nutrient components and energy values for both age groups are presented in Table 7 and Table 8. For 12–14-day-old broilers, PA was significantly negatively correlated with AME, AMEn, NE, AME/GE, and AMEn/GE (*p* < 0.05). DM was significantly positively correlated with NE/AMEn. CP was highly significantly positively correlated with AMEn and significantly positively correlated with NE/AMEn (*p* < 0.05). For 26–28-day-old broilers, GE was significantly positively correlated with NE/AME (*p* < 0.05). EE was significantly negatively correlated with AMEn/GE (*p* < 0.05). CF was significantly positively correlated with AME/GE (*p* < 0.05). Ash was significantly negatively correlated with AME, AMEn, AME/GE, and AMEn/GE (*p* < 0.05).

Based on the chemical composition and energy values of Corn 1–10, multiple linear stepwise regression was used to construct prediction equations for AME, AMEn, and NE. As shown in Table 9, for 12–14-day-old broilers, 2 equations each were established for AME and AMEn, 4 for NE, with R^2^ values ranging from 0.524 to 0.972, indicating good fit. As shown in Table 10, for 26–28-day-old broilers, 2 equations for AME, 2 for AMEn, and 3 for NE were established, with R^2^ values ranging from 0.549 to 0.946, demonstrating good fit.

### 3.4. Validation of the Prediction Equations

**Figure 1:**
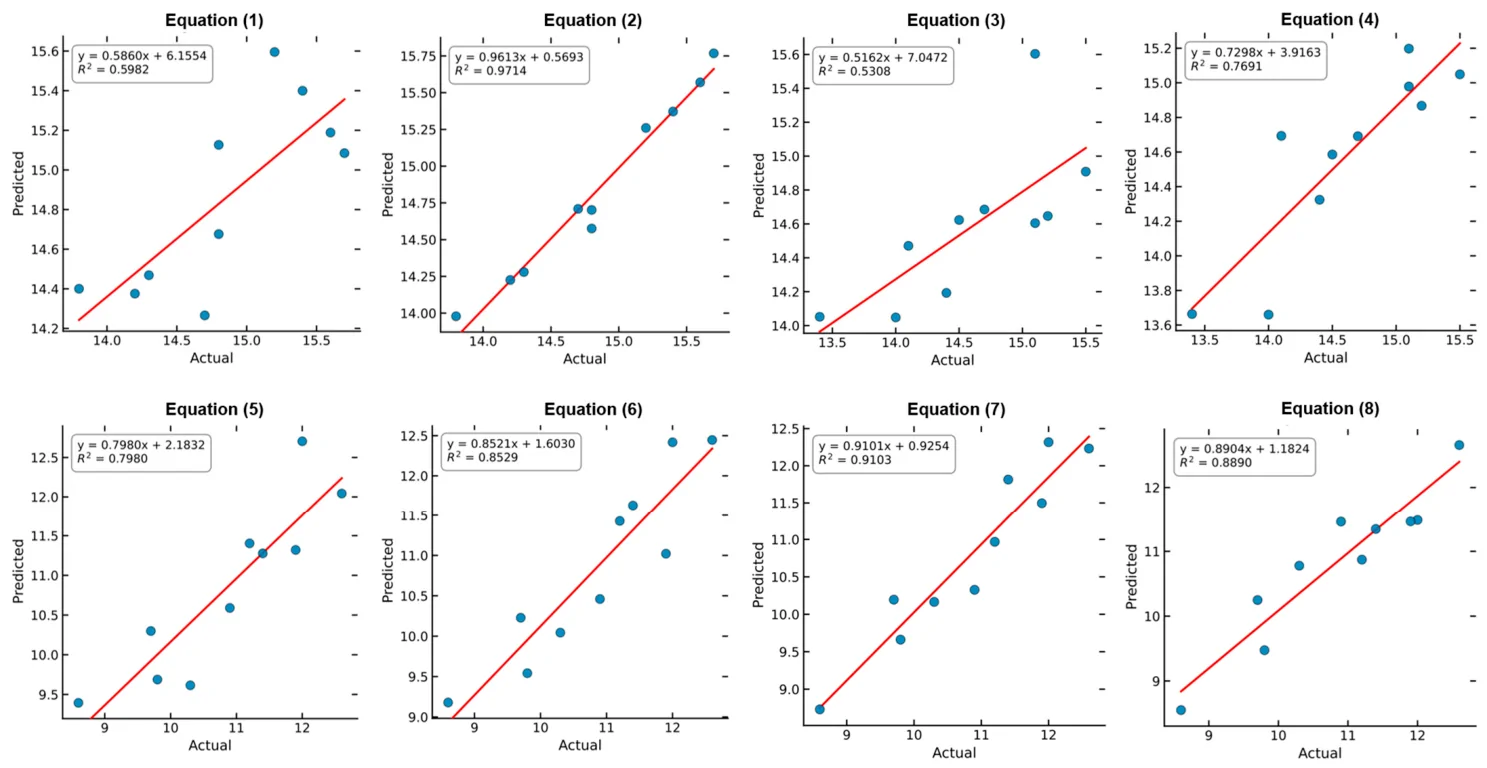
Relationship between predicted and measured corn energy values in 12-to-14-day-old broilers.

**Figure 2:**
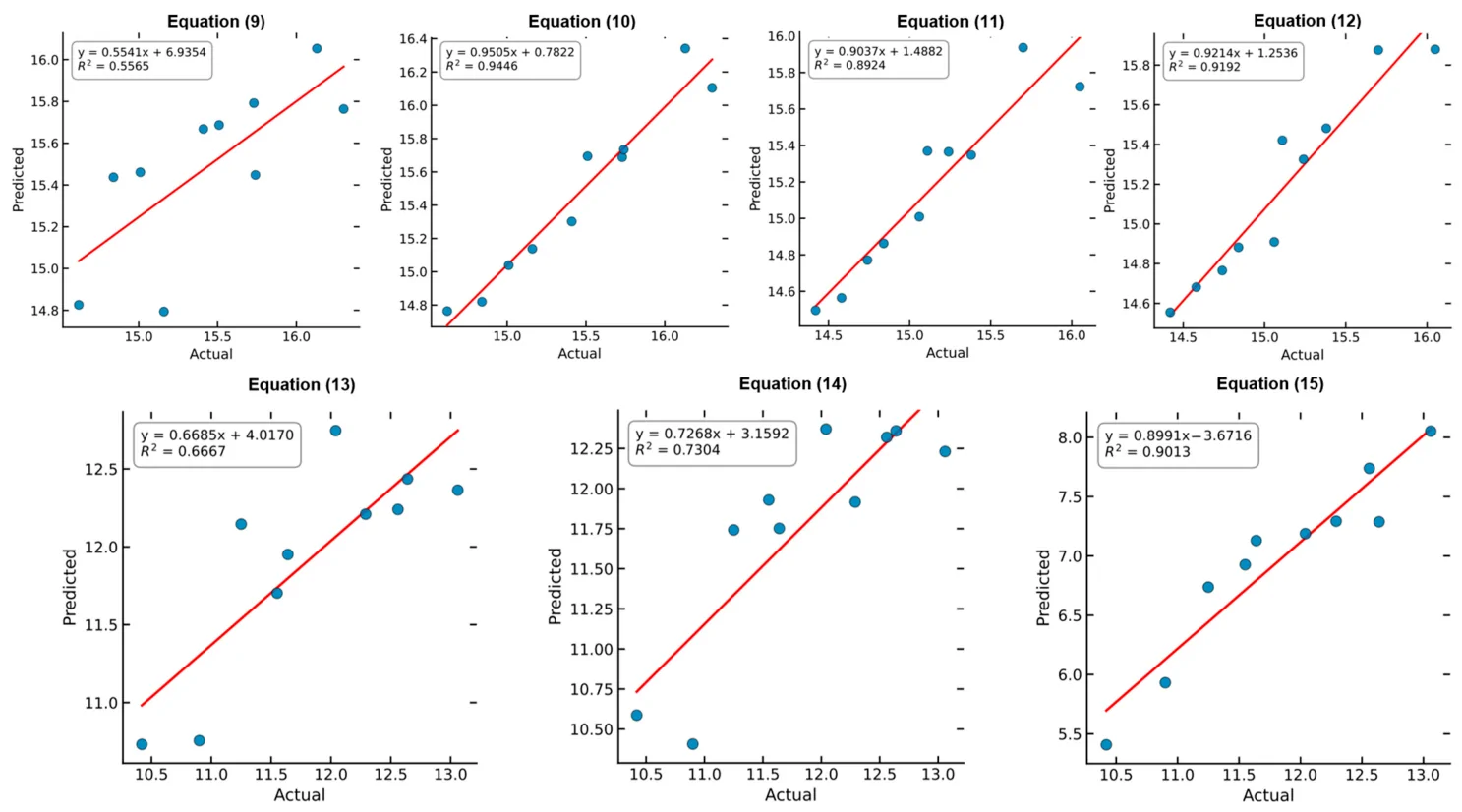
Relationship between predicted and measured corn energy values in 26-to-28-day-old broilers.

To evaluate the predictive performance of the established equations, the same corn sample data used for model development were re-substituted into each equation, and the predicted energy values were compared with the measured values. The validation results are presented in Figure 1 and Figure 2. As shown in Figure 1, for 12–14-day-old broilers, the predicted values of AME, AMEn, and NE were generally in good agreement with the measured values, and the equations adequately captured the overall variation in energy content. For 26–28-day-old broilers (Figure 2), the predictions were also satisfactory, with generally higher precision and stability, as well as lower bias and better reproducibility, compared with those for the younger birds. In summary, the re-substitution validation confirms that the equations developed in this study exhibit good predictive performance and can be used for rapid estimation of corn energy values; however, since the validation used the same dataset as model calibration, further validation with independent data is warranted to confirm their external applicability.

## 4. Discussion

In the present study, the chemical compositions of the 10 corn varieties differed considerably. The CVs of CP, EE, NDF, ADF, Ash, CF, and PA all exceeded 10%, confirming that the selected corn samples were representative for determining available energy values. Differences in nutrient composition among corn varieties may be attributed to variations in geographic origin, temperature, and harvest time [17]. Furthermore, the chemical composition values in this study were comparable to those reported by Guo et al. [12], suggesting that the chemical profiles of our corn samples were within a reasonable range relative to previous studies. Overall, the substantial and justifiable variation in chemical composition among the selected corn samples provided a reliable database for the accurate determination of available energy values and the subsequent development of prediction equations.

The 10 corn samples in this study exhibited considerable variability in AME, AMEn, and NE values. The ranges of AME, AMEn, and NE measured in our study encompassed those reported by Liu et al. [18] for corn (AME: 14.59–15.38 MJ/kg; AMEn: 14.45–15.05 MJ/kg; NE: 10.78–11.27 MJ/kg). Differences in energy values among corn varieties are mainly influenced by genetic factors, grain texture, climatic and soil conditions of the growing region, and harvesting and storage conditions [19]. Importantly, variations in chemical composition across different varieties and origins ultimately affect energy values. Our study further revealed that, in 12–14-day-old broilers, PA was significantly negatively correlated with CP and EE. This may be because phytic acid forms insoluble complexes with CP and EE in the digestive tract, and it also directly binds to endogenous digestive enzymes, inhibiting their activity [20]. Consequently, PA negatively affects AME and NE not only by directly reducing nutrient digestibility but also indirectly by impairing the effective utilization of CP and EE [21], which is consistent with previous reports on the anti-nutritional effects of PA [22]. In 26–28-day-old broilers, Ash was significantly negatively correlated with AME and AMEn, which aligns with the findings of Rochell [23].

We also observed considerable variation in energy utilization efficiency among corn varieties. The average AME/GE ratios in our study were 79% and 82%, which are similar to the 78% reported by Cerrate [24] and the 79% reported by Tay-Zar [25]. The NE/AME ratio reflects the efficiency of converting AME to NE, and its magnitude is directly influenced by HI. HI mainly originates from the heat produced during nutrient metabolism, including digestion, absorption, and metabolic processes [26]. Our mean NE/AME values of 73% and 76% closely matched previous findings, which reported NE/AME averages ranging from 73% to 80% [25,27,28]. Additionally, we found that GE was significantly positively correlated with NE/AME, while PA and Ash were significantly negatively correlated with AME/GE and AMEn/GE. This may be because GE is positively correlated with major energy-yielding nutrients; thus, ingredients with higher GE often contain more readily metabolizable components, thereby improving energy utilization efficiency [29]. PA acts as an anti-nutritional factor in broilers, hindering nutrient utilization and reducing energy conversion efficiency [30]. Ash represents the total mineral content, which contains no energy; an increase in Ash may dilute other energy-yielding components, leading to reduced energy conversion efficiency [23]. Taken together, these findings indicate that the energy utilization efficiency of corn is synergistically regulated by its chemical composition.

Interestingly, our results showed that NE values and AME/GE were significantly higher, and AMEn/GE tended to be higher, in 26–28-day-old broilers compared with 12–14-day-old broilers. This finding is consistent with Liu et al. [31], who evaluated five corn samples in broilers at 14–16 days (starter phase) and 28–30 days (grower phase). They reported that AMEn increased from 16.25–17.41 to 17.23–17.60 MJ/kg DM, and NE increased from 9.93–11.67 to 11.19–12.27 MJ/kg, indicating improved nutrient digestibility and energy values with advancing broiler age. Broiler strain, age, and body weight can all influence dietary energy utilization [32,33]. The physiological basis for the age effect mainly involves the maturation of the digestive system [34]. As broilers age, intestinal surface area increases and digestive enzyme secretion capacity improves, thereby enhancing the digestion and absorption of starch, fat, and protein from corn [35,36]. Frank et al. reported that AME in corn–soybean meal-based diets increased with age, attributable to improved utilization capacity for starch, fat, and protein [37]. In addition, as broilers mature and their digestive tract develops, the passage rate of digesta slows, allowing more time for nutrient digestion and absorption [38]. Similarly, Song et al. confirmed that ME and ME/GE of corn were significantly higher in 25–28-day-old broilers than in 11–14-day-old broilers [39]. In conclusion, broiler age significantly affects corn energy values. Therefore, age-specific energy values should be used when formulating diets for different growth stages to achieve precision nutrition and high production efficiency.

Based on the chemical composition and energy values of the 10 corn samples, we developed prediction equations for AME, AMEn, and NE using multiple linear stepwise regression for broilers at different ages. Regarding variable selection, Ash consistently acted as a negative predictor in almost all equations, which aligns with the findings of Liu et al. [31] and Rochell et al. [23], indicating a universal negative impact of Ash on energy utilization. The sign of PA varied across energy metrics: it was mostly positive in AME equations, mostly negative in AMEn equations, and mixed in NE equations, suggesting that PA may affect different energy fractions through distinct metabolic pathways, though its anti-nutritional effect remains non-negligible. CP and ADF were retained in multiple models, with CP showing positive coefficients in some equations and ADF generally negative, consistent with reports by Ning et al. [40] and Tay-Zar et al. [25]. Interestingly, EE exhibited positive coefficients in the AME and NE equations for 12–14-day-old birds but turned negative in NE Equation (7) for 26–28-day-old birds, implying that age may influence the energy contribution of fat. NDF appeared only in a few equations, while ST was selected as a positive predictor in isolated cases, reflecting the compositional variation in corn samples. Compared with the corn co-product models of Rochell et al. [23], our whole-corn models did not include hemicellulose and had lower frequency of EE inclusion, likely due to the compositional differences between whole corn and co-products. In terms of goodness-of-fit, the R^2^ values of our equations ranged from 0.524 to 0.972, which are comparable to or better than those reported in the literature (0.66–0.96), indicating satisfactory explanatory power.

From a practical standpoint, the prediction equations developed in this study offer a rapid and cost-effective tool for the feed industry. By measuring only routine chemical components, nutritionists can estimate corn AME, AMEn, and NE values without conducting time-consuming and expensive animal metabolism trials. This enables feed mills to dynamically adjust energy values in formulation software during raw material procurement, thereby improving diet accuracy and lowering feed costs. The equations also facilitate on-site quality evaluation of incoming corn batches, supporting precision nutrition and sustainable poultry production.

## 5. Conclusions

This study determined the AME, AMEn, and NE values of 10 corn samples in AA broilers at 12–14 and 26–28 days of age and established prediction equations based on proximate nutrient composition. Broiler age significantly affected energy utilization, with higher NE and AME/GE ratios in older birds. This finding implies that age-specific energy values should be applied when formulating diets for different growth stages. The prediction equations exhibited good goodness-of-fit, with high R^2^ values, demonstrating their reliability for estimating corn energy values from routine chemical analyses. These equations provide a practical and cost-effective tool for feed formulation and ingredient quality evaluation. Future validation with a larger number of corn samples is recommended to further confirm their generalizability.

## Figures and Tables

**Table 1:** Conventional chemical composition of corn materials (dry matter basis, %).

Items	Corn										Mean	CV
	1	2	3	4	5	6	7	8	9	10		
GE (MJ/kg)	18.74	18.70	18.46	18.95	18.62	18.49	18.84	18.82	18.81	18.94	18.74	0.91
DM	88.33	87.60	88.39	86.11	86.11	87.12	87.70	85.87	87.35	86.79	87.14	1.04
CP	8.89	8.42	7.56	10.85	10.93	7.77	7.80	8.11	9.52	10.50	9.03	14.67
EE	3.27	3.38	3.58	3.83	4.13	2.87	4.04	3.51	3.31	4.12	3.60	11.65
CF	2.29	2.09	2.26	1.88	2.66	1.42	1.82	1.42	2.01	1.86	1.97	19.42
NDF	8.41	9.21	9.29	9.57	11.64	8.21	10.99	9.01	10.21	14.20	10.07	17.90
ADF	2.22	2.34	2.50	2.39	3.04	1.26	3.18	2.90	2.88	3.39	2.61	23.46
Ash	1.17	1.23	1.37	1.43	1.15	1.44	1.33	1.46	1.27	1.79	1.36	13.58
PA	8.92	8.39	9.24	5.86	5.86	9.03	7.77	10.26	8.65	9.51	8.35	17.55
ST	67.02	77.65	66.79	78.79	70.41	72.60	78.08	75.23	79.66	79.29	74.55	6.74

GE, gross energy; DM, dry matter; CP, crude protein; EE, ether extract; CF, crude fiber; NDF, neutral detergent fiber; ADF, acid detergent fiber; Ash, crude ash; PA, phytic acid; ST, Starch; CV, coefficients of variation.

**Table 2:** Composition of reference and test diets for broilers (%, air-dry basis).

Ingredient	12–14 d		26–28 d	
	Reference Diet	Test Diet	Reference Diet	Test Diet
Corn	56.56	33.10	60.03	35.22
Soybean meal	24.65	14.41	21.26	12.48
Corn gluten meal	3.10	1.81	2.10	1.23
Peanut meal	4.30	2.51	4.10	2.41
Soybean oil	3.05	1.78	4.07	2.39
DDGS ^1^	3.30	1.93	3.90	2.29
Test corn	0.00	40.00	0.00	40.00
L-lysine HCl (70%)	1.00	0.58	0.96	0.56
DL-methionine (99%)	0.25	0.15	0.24	0.14
L-threonine (99%)	0.14	0.08	0.15	0.09
L-tryptophan	0.03	0.03	0.00	0.00
Dicalcium phosphate	1.14	1.14	0.70	0.70
Sodium chloride	0.25	0.25	0.24	0.24
Choline chloride (60%)	0.11	0.11	0.11	0.11
Sodium bicarbonate	0.12	0.12	0.12	0.12
Limestone	0.90	0.90	0.92	0.92
Vitamin and mineral Premix ^2^	0.50	0.50	0.50	0.50
Phytase	0.20	0.20	0.20	0.20
Titanium dioxide	0.40	0.40	0.40	0.40
Total	100.00	100.00	100.00	100.00

^1^ DDGS, dried distillers grains with solubles. ^2^ Premix provided the following per kg of the diet: vitamin A 12,500 IU; vitamin D_3_ 3500 IU; vitamin E 20 IU; vitamin K 3 mg; vitamin B_1_ 0.01 mg; vitamin B_2_ 8.00 mg; vitamin B_6_ 4.5 mg; vitamin B_12_ 0.02 mg; nicotinic acid 34 mg; pantothenic acid 12 mg; folic acid 0.5 mg; biotin 0.2 mg; Fe 80 mg; Cu 8 mg; Zn 80 mg; Mn 80 mg; I 0.7 mg; Se 0.3 mg.

**Table 3:** Nutrient levels of the reference diet and test diets for broilers (air-dry basis).

Items	Reference Diet	Test Diets									
		1	2	3	4	5	6	7	8	9	10
12–14 d											
DM, %	89.56	89.69	89.16	88.88	88.63	90.11	89.25	90.66	90.02	90.24	89.53
GE, MJ/kg	18.67	18.16	18.21	18.17	18.44	19.03	18.32	18.77	18.88	18.65	18.85
CP, %	24.09	18.30	18.54	18.98	17.57	17.98	17.59	17.53	17.70	17.49	18.25
26–28 d											
DM, %	87.03	87.4	87.36	86.33	87.56	86.75	86.93	89.80	89.29	90.01	89.41
GE, MJ/kg	19.18	18.64	18.62	18.83	18.79	18.82	18.83	18.60	18.58	18.85	19.00
CP, %	21.94	17.13	16.55	16.59	16.19	16.58	16.48	16.08	16.61	15.94	16.89

DM, dry matter; GE, gross energy; CP, crude protein.

**Table 4:** Energy values and metabolizable energy utilization of corn for 12-to-14-day-old broilers (DM basis).

Items	Corn										Mean	SEM	*p*-Value
	1	2	3	4	5	6	7	8	9	10			
AME, MJ/kg	15.44	15.66	13.84	15.21	15.59	14.82	14.78	14.34	14.25	14.66	14.86	0.157	0.122
AMEn, MJ/kg	15.09 ^ab^	15.21 ^ab^	13.39 ^b^	15.10 ^ab^	15.35 ^a^	14.69 ^ab^	14.54 ^ab^	14.04 ^ab^	13.98 ^ab^	14.39 ^ab^	14.58	0.163	0.044
NE, MJ/kg	12.56	11.99	10.28	10.90	11.39	11.19	11.88	9.67	8.61	9.75	10.82	0.277	0.062
AME/GE, %	82.42	83.76	74.99	80.29	83.73	80.15	78.45	76.21	75.76	77.37	79.31	0.844	0.122
AMEn/GE, %	80.55 ^ab^	81.32 ^ab^	72.52 ^b^	79.70 ^ab^	83.41 ^a^	79.44 ^ab^	77.15 ^ab^	74.59 ^ab^	74.32 ^ab^	75.97 ^ab^	77.93	0.875	0.044
NE/AME, %	81.32	76.57	72.94	71.73	72.90	75.48	80.30	67.21	60.37	66.49	72.53	1.457	0.070
NE/AMEn, %	83.22	78.87	75.43	72.26	73.23	76.17	81.65	68.68	61.55	67.71	73.87	1.496	0.063

Different letters indicate significant differences (*p* < 0.05). AME, apparent metabolizable energy; AMEn, nitrogen-corrected AME; NE, net energy; GE, gross energy. SEM, standard error of the mean.

**Table 5:** Energy values and metabolizable energy utilization of corn for 26 to 28-day-old broilers (DM basis).

Items	Corn										Mean	SEM	*p*-Value
	1	2	3	4	5	6	7	8	9	10			
AME, MJ/kg	16.13 ^ab^	15.73 ^ab^	15.41 ^ab^	14.62 ^b^	15.74 ^ab^	15.01 ^ab^	14.84 ^ab^	15.51 ^ab^	16.30 ^a^	15.16 ^ab^	15.45	0.121	0.023
AMEn, MJ/kg	15.70 ^ab^	15.38 ^ab^	15.06 ^ab^	14.42 ^b^	15.24 ^ab^	14.74 ^ab^	14.58 ^ab^	15.11 ^ab^	16.05 ^a^	14.84 ^ab^	15.11	0.119	0.048
NE, MJ/kg	12.29	11.64	10.42	12.04	11.55	10.90	12.64	11.25	13.06	12.56	11.83	0.334	0.844
AME/GE, %	86.05 ^a^	84.11 ^ab^	83.49 ^ab^	77.15 ^b^	84.56 ^ab^	81.19 ^ab^	78.76 ^ab^	82.43 ^ab^	86.69 ^a^	80.03 ^ab^	82.45	0.659	0.010
AMEn/GE, %	83.80 ^ab^	82.25 ^ab^	81.58 ^ab^	76.09 ^b^	81.85 ^ab^	79.71 ^ab^	77.36 ^ab^	80.30 ^ab^	85.35 ^a^	78.33 ^ab^	80.66	0.646	0.026
NE/AME, %	76.27	74.01	67.71	83.18	73.16	72.59	85.24	72.10	79.86	82.69	76.68	2.092	0.769
NE/AMEn, %	78.34	75.67	69.29	84.28	75.88	73.94	86.79	73.95	81.15	84.50	78.38	2.155	0.828

Different letters indicate significant differences (*p* < 0.05). AME, apparent metabolizable energy; AMEn, nitrogen-corrected AME; NE, net energy; GE, gross energy. SEM, standard error of the mean.

**Table 6:** Energy value and energy utilization of corn in broilers of different days of age (%, DM basis).

Items	12–14 Days	26–28 Days	SEM	*p*-Value
Energy values, MJ/kg				
AME, MJ/kg	14.86	15.45	0.174	0.280
AMEn, MJ/kg	14.58	15.11	0.149	0.118
NE, MJ/kg	10.82	11.83	0.256	0.045
Energy utilization, %				
AME/GE, %	79.31	82.45	0.784	0.042
AMEn/GE, %	77.93	80.66	0.775	0.078
NE/AME, %	72.53	76.68	1.416	0.147
NE/AMEn, %	73.84	78.38	1.442	0.118

AME, apparent metabolizable energy; AMEn, nitrogen-corrected AME; NE, net energy; GE, gross energy. SEM, standard error of the mean.

**Table 7:** Correlation analysis of nutritional components, energy values, and energy utilization efficiency for corn samples in broilers between 12 and 14 days of age.

	GE	DM	CP	EE	NDF	ADF	CF	Ash	PA	ST	AME	AMEn	NE	AME/GE	AMEn/GE	NE/AME	NE/AMEn
GE	1																
DM	−0.316 *	1															
CP	0.439 **	−0.578 **	1														
EE	0.449 **	−0.360 *	0.596 **	1													
NDF	0.420 **	−0.300	0.602 **	0.817 **	1												
ADF	0.573 **	−0.235	0.437 **	0.846 **	0.782 **	1											
CF	−0.137	0.136	0.522 **	0.510 **	0.357*	0.410 **	1										
Ash	0.303*	−0.202	−0.069	0.029	0.295	0.024	−0.641 **	1									
PA	−0.122	0.389 **	−0.699 **	−0.550 **	−0.291	−0.192	−0.568 **	0.444 **	1								
ST	0.719 **	−0.338 *	0.202	0.170	0.336 **	0.303 **	−0.423 **	0.404 **	−0.052	1							
AME	0.036	−0.127	0.251	0.118	0.029	−0.060	0.229	−0.255	−0.347 **	−0.057	1						
AMEn	0.033	−0.219	0.327 **	0.156	0.068	−0.058	0.238	−0.259	−0.431 **	−0.051	0.983 **	1					
NE	−0.135	0.158	0.071	0.023	−0.144	−0.191	0.158	−0.260	−0.204 **	−0.260	0.738 **	0.710 **	1				
AME/GE	−0.087	−0.089	0.197	0.062	−0.022	−0.129	0.224	−0.291	−0.331*	−0.143	0.992 **	0.976 **	0.752 **	1			
AMEn/GE	−0.084	−0.183	0.275	0.102	0.020	−0.124	0.252	−0.293	−0.414 **	−0.134	0.975 **	0.993 **	0.724 **	0.983 **	1		
NE/AME	−0.181	0.250	−0.211	−0.025	−0.195	−0.222	0.074	−0.196	−0.099	−0.293	0.485 **	0.460 **	0.947 **	0.506 **	0.480 **	1	
NE/AMEn	−0.182	0.302 *	−0.256	−0.047	−0.216	−0.220	0.065	−0.187	−0.046	−0.296	0.465 **	0.422 **	0.937 **	0.486 **	0.442 **	0.995 **	1

*, ** represent *p* < 0.05 and *p* < 0.01, respectively. GE, gross energy; DM, dry matter; CP, crude protein; EE, ether extract; CF, crude fiber; NDF, neutral detergent fiber; ADF, acid detergent fiber; Ash, crude ash; PA, phytic acid; ST, starch; AME, apparent metabolizable energy; AMEn, nitrogen-corrected AME; NE, net energy.

**Table 8:** Correlation analysis of nutritional components, energy values, and energy utilization efficiency for corn samples in broilers between 26 and 28 days of age.

	GE	DM	CP	EE	NDF	ADF	CF	Ash	PA	ST	AME	AMEn	NE	AME/GE	AMEn/GE	NE/AME	NE/AMEn
GE	1																
DM	−0.335 *	1															
CP	0.436 **	−0.499 **	1														
EE	0.412 **	−0.401 *	0.591 **	1													
NDF	0.454 **	−0.299 *	0.624 **	0.808 **	1												
ADF	0.569 **	−0.320 *	0.433 **	0.809 **	0.779 **	1											
CF	−0.286 *	0.265	0.444 **	0.335 *	0.182	0.186	1										
Ash	0.457 **	−0.297 *	0.118	0.288 *	0.552 **	0.294 *	−0.589 **	1									
PA	0.024	0.264	−0.596 **	−0.440 **	−0.138	−0.048	−0.578 **	0.444 **	1								
ST	0.701 **	−0.360 *	0.231	0.203	0.410 **	0.380 **	−0.460 **	0.453 **	0.009	1							
AME	−0.111	0.183	0.007	−0.216	−0.122	0.017	0.266	−0.334 *	0.103	−0.154	1						
AMEn	−0.075	0.197	−0.004	−0.239	−0.127	0.001	0.216	−0.300 *	0.110	−0.090	0.977 **	1					
NE	0.233	0.028	0.136	0.069	0.136	0.147	0.024	0.019	−0.031	0.177	0.279 *	0.268	1				
AME/GE	−0.248	0.225	−0.052	−0.267	−0.182	−0.062	0.302 *	−0.390 *	0.095	−0.249	0.990 **	0.963 **	0.240	1			
AMEn/GE	−0.213	0.240	−0.064	−0.291 *	−0.187	−0.078	0.253	−0.359 **	0.103	−0.186	0.973 **	0.990 **	0.230	0.978 **	1		
NE/AME	0.280 *	−0.022	0.144	0.140	0.175	0.144	−0.049	0.117	−0.076	−0.230	−0.013	−0.016	0.955 **	−0.051	−0.054	1	
NE/AMEn	0.265	−0.027	0.149	0.148	0.178	0.148	−0.031	0.105	−0.080	0.208	−0.005	−0.024	0.955 **	−0.042	−0.060	0.997 **	1

*, ** represent *p* < 0.05 and *p* < 0.01, respectively. GE, gross energy; DM, dry matter; CP, crude protein; EE, ether extract; CF, crude fiber; NDF, neutral detergent fiber; ADF, acid detergent fiber; Ash, crude ash; PA, phytic acid; ST, starch; AME, apparent metabolizable energy; AMEn, nitrogen-corrected AME; NE, net energy.

**Table 9:** Prediction equations for energy values of corn in 12 to 14-day-old broilers (DM basis).

Equation NO.	Prediction Equation	R^2^	RSE
(1)	AME = −1.417 × ADF − 2.680 × Ash + 0.136 × PA + 2.423 × GE + 1.884 × EE − 31.111	0.609	0.573
(2)	AME = −3.981 × ADF − 8.370 × Ash + 1.293 × PA + 0.644 × CP + 6.284 × EE + 0.165 × ST − 14.912	0.972	0.178
(3)	AMEn = 1.243 × GE − 0.437 × ADF − 0.267 × PA − 5.335	0.524	0.537
(4)	AMEn = 2.509 × GE + 0.345 × NDF − 2.733 × Ash − 1.221 × ADF − 0.074 × PA − 28.381	0.730	0.945
(5)	NE = 1.943 × AME − 0.512 × CP − 13.412	0.799	0.625
(6)	NE = 1.869 × AME − 0.432 × CP − 0.060 × ST − 8.556	0.853	0.577
(7)	NE = 1.946 × AME − 0.585 × CP − 0.066 × ST + 0.837 × EE − 10.936	0.909	0.499
(8)	NE = 4.969 × GE + 1.135 × PA − 9.025 × Ash + 8.054 × EE − 5.258 × ADF − 94.735	0.892	0.605

CP, crude protein; CF, crude fiber; NDF, neutral detergent fiber; Ash, crude ash; PA, phytic acid; AME, apparent metabolizable energy; AMEn, nitrogen-corrected AME; NE, net energy.

**Table 10:** Prediction equations for energy values of corn in 26 to 28-day-old broilers (DM basis).

Equation NO.	Prediction Equation	R^2^	RSE
(9)	AME = −2.23 × Ash + 0.21 × PA + 16.78	0.549	0.360
(10)	AME = −3.37 × Ash + 0.47 × PA + 0.38 × CP + 12.70	0.946	0.144
(11)	AMEn = −3.02 × Ash + 0.34 × CP + 0.43 × PA + 12.60	0.897	0.199
(12)	AMEn = −3.27 × Ash + 0.33 × CP + 0.44 × PA + 0.02 × ST + 11.49	0.921	0.190
(13)	NE = 4.32 × GE − 1.38 × Ash − 67.13	0.668	0.544
(14)	NE = 3.83 × GE − 1.92 × Ash + 0.14 × NDF − 58.79	0.733	0.527
(15)	NE = 4.49 × GE − 2.77 × Ash − 1.52 × EE + 0.44 × NDF − 0.01 × PA − 72.25	0.902	0.391

GE, gross energy; CP, crude protein; NDF, neutral detergent fiber; EE, ether extract; Ash, crude ash; PA, phytic acid; AME, apparent metabolizable energy; AMEn, nitrogen-corrected AME; NE, net energy.

## Data Availability

The data that support the findings of this study are not publicly available due to ongoing research and will be made available upon reasonable request from the corresponding author.

## Abbreviations

The following abbreviations are used in this manuscript:

AA	Arbor Acres
ADF	acid detergent fiber
AME	apparent metabolizable energy
AMEn	nitrogen-corrected apparent metabolizable energy
Ash	crude ash
CF	crude fiber
CP	crude protein
CV	coefficient of variation
DDGS	dried distillers grains with solubles
DM	dry matter
EE	ether extract
GE	gross energy
HI	heat increment
ME	metabolizable energy
NDF	neutral detergent fiber
NE	net energy
NSP	non-starch polysaccharide
HP	heat production
RQ	respiratory quotient
SEM	standard error of the mean
